# Effects of *Yarrowia lipolytica* supplementation on growth performance, intestinal health and apparent ileal digestibility of diets fed to nursery pigs

**DOI:** 10.5713/ab.21.0369

**Published:** 2021-10-29

**Authors:** Yi-Chi Cheng, Marcos Elias Duarte, Sung Woo Kim

**Affiliations:** 1 Department of Animal Science, North Carolina State University, Raleigh, NC 27695, USA

**Keywords:** Apparent Ileal Digestibility, Growth Performance, Intestinal Health, Nursery Pigs, *Yarrowia lipolytica*, Yeast

## Abstract

**Objective:**

The objective was to evaluate the efficacy of increasing supplementation of *Yarrowia lipolytica* (YL) up to 3.0% replacing 1.6% poultry fat and 0.9% blood plasma for growth performance, intestinal health and nutrient digestibility of diets fed to nursery pigs.

**Methods:**

Twenty-four pigs weaned at 24 d of age (initial body weight at 7.2±0.6 kg) were allotted to three dietary treatments (n = 8) based on the randomized complete block. The diets with supplementation of YL (0.0%, 1.5%, and 3.0%, replacing poultry fat and blood plasma up to 1.6% and 0.9%, respectively) were fed for 21 d. Feed intake and body weight were recorded at d 0, 10, and 21. Fecal score was recorded at every odd day from d 3 to 19. Pigs were euthanized on d 21 to collect proximal and distal jejunal mucosa to measure intestinal health markers including tumor necrosis factor-alpha, interleukin-8, immunoglobulin A and immunoglobulin G. Ileal digesta was collected for apparent ileal digestibility (AID) of nutrients in diets. Data were analyzed using Proc Mixed of SAS.

**Results:**

Supplementation of YL (1.5% and 3.0%) replacing poultry fat and blood plasma did not affect growth performance, fecal score and intestinal health. Supplementation of YL at 1.5% did not affect nutrient digestibility, whereas supplementation of YL at 3.0% reduced AID of dry matter (40.2% to 55.0%), gross energy (44.0% to 57.5%), crude protein (52.1% to 66.1%), and ether extract (50.8% to 66.9%) compared to diets without supplementation.

**Conclusion:**

*Yarrowia lipolytica* can be supplemented at 1.5% in nursery diets, replacing 0.8% poultry fat and 0.45% blood plasma without affecting growth performance, intestinal health and nutrient digestibility. Supplementation of YL at 3.0% replacing 1.6% poultry fat and 0.9% blood plasma did not affect growth performance and intestinal health, whereas nutrient digestibility was reduced.

## INTRODUCTION

Animal fats and plant oils have been used in swine diets in order to improve dietary energy digestibility [1], growth and feed efficiency [2]. Fatty acids (FA) play important roles in cell-signaling, immune function and cell membrane integrity [3]. In addition, FA can be divided into two main groups comprised of saturated FA and unsaturated FA. The quantities of FA vary greatly, depending on the sources (Table 1). Unsaturated FA improves the animal health and meat quality by enhancing intestinal barrier functions [4] and altering the pork fat composition [5], respectively.

The production of animal fats was increased by 15% from 2013 to 2020 in the USA [6]. Other than animal fats, the production of plant oils, including soybean oil, seed oils, palm oil and coconut oil, was increased by 22% from 172 to 209 million tons globally during the same period [6]. However, animal fats and plant oils have also been largely used in different industry sectors, including biodiesel and food industries [7]. For the bioenergy production, the global demand of fats and oils has been increased by 38% in the last decade [8] and the food industry requires higher fat quality than the feed industry. Therefore, fats with lower quality, such as restaurant grease and animal-vegetable blend oils are commonly used in animal production in the USA. These fat by-products could have higher peroxidation possibly with negative impacts on intestinal health and growth performance of animals [9]. Due to these concerns, alternative fat supplements have been sought, including oleaginous microorganisms [10] and insect oils [11].

Oleaginous microorganisms include yeasts, bacteria and microalgae rich in oil and these are also called single cell oils (SCO). Depending on the sources, SCO contain FAs with unique profiles different from conventional fats and oils, proteins from cell wall with unique functions and other organic compounds such as vitamins [12]. Recently SCO have been used in aquaculture to replace conventional fats without adverse effects on fish growth and fish quality [13]. *Yarrowia lipolytica* is one of oleaginous yeasts and generally recognized as safe in food industry [14]. The strain of *Yarrowia lipolytica* used in this study contains 72.7% of fat and high concentration (52.3%) of oleic acid (Table 1). Oleic acid has been shown to reduce oxidative stress and inflammatory response by activating peroxisome proliferator activated receptor in animals [15]. Previous studies showed that animal fats and plant oils are 80% digestible by weaned pigs [1]. Similar to *Yarrowia lipolytica*, restaurant grease has similar oleic acid content, however, its high peroxidation rate compromises its benefits [9].

In general, β-glucans, including 1,3 and 1,6 β-glucans, are the major components of the yeast cell wall (55% to 65%) [16]. The second major component from the yeast cell wall is mannoprotein (30% to 40%), which is glycoprotein mainly containing mannans on the external surface of the wall [17]. Beta-glucans and mannans are bioactive compounds from yeast cell walls with potential benefits on the development of intestinal immune system of animals [18,19] that can be similar to the role of functional compounds in blood plasma. Blood plasma has long been used in swine industry in order to improve growth performance [20] of nursery pigs by enhanced intestinal health that reduces inflammatory activation and promotes the intestinal barrier function [21]. Otherwise, β-glucans and mannans from yeast cell wall also have beneficial effects on intestinal health by mitigating the release of pro-inflammatory cytokines and preventing the colonization of pathogens on intestinal mucosa [18,19].

Therefore, the hypothesis was that *Yarrowia lipolytica* can be used as a fat supplement in nursery diets providing bioactive components, including oleic acid, β-glucans and mannans to enhance intestinal health, nutrient digestibility and growth performance of nursery pigs. To test the hypothesis, the objective was to evaluate the efficacy of increasing supplementation of *Yarrowia lipolytica* up to 3.0% replacing 1.6% poultry fat and 0.9% blood plasma for growth, intestinal health and digestibility of nutrients in diets fed to nursery pigs.

## MATERIALS AND METHODS

### Animal care

The experimental protocol was approved by the Institutional Animal Care and Use Committee of North Carolina State University.

### Animal, design and diets

Twenty-four newly weaned pigs at 24 d of age with initial body weight (BW) of 7.2±0.6 kg were allotted to three dietary treatments (n = 8) based on the randomized complete block design. Initial BW (light and heavy) and sex (gilts and barrows) were considered as blocks following Holanda et al [22]. Pigs were housed in pens individually. The dietary treatments consisted of a basal diet supplemented with *Yarrowia lipolytica* (CJ Bio; Fort Dodge, IA, USA) at three levels (0.0%, 1.5%, and 3.0% of *Yarrowia lipolytica* as YL0, YL1.5, and YL3, respectively). *Yarrowia lipolytica* was supplemented at 1.5% and 3.0%, replacing poultry fat (0.8% and 1.6%, respectively) and blood plasma (0.45% and 0.9%, respectively) balancing metabolizable energy and standardized ileal digestible Lys in phase 1 and phase 2 diets. The nutrient compositions of *Yarrowia lipolytica* and experimental diets are shown in Table 1 and 2, respectively. All experimental diets were formulated to meet or exceed the nutrient requirements suggested by NRC [23] and fed to pigs for 21 d divided into two phases: phase 1 (d 0 to 10) and phase 2 (d 10 to 21). Titanium dioxide at 0.4% was added in diets as an indigestible external marker during the last 7 d of experiment.

### Growth performance and fecal score

Body weight and feed intake were recorded at d 0, 10, and 21 to calculate average BW, average daily gain (ADG), average daily feed intake (ADFI), and gain to feed ratio (G:F). Fecal scores from every pig were recorded every odd day using a 1 to 5 scale: i) very hard and dry stool, ii) firm stool, iii) normal stool, iv) loose stool, and v) watery stool with no shape following Guo et al [25].

### Sample collection and processing

After 21 d of feeding, all pigs were euthanized by the penetration of a captive bolt to the head following by exsanguination in order to collect samples. Sections of proximal (1.5 m after the pyloric duodenal junction) and distal (1.5 m before the ileocecal junction) jejunum were collected and rinsed with 0.9% saline solution. The jejunal mucosa was collected into 2 mL microcentrifuge tubes and immediately frozen in liquid nitrogen and then stored at −80°C for immune status measurements. Ileal digesta was collected into 150 mL containers placed on ice and then stored at −20°C for apparent ileal digestibility (AID).

Jejunal mucosa samples (1 g) from the 2 mL microcentrifuge tubes were taken and added with 1 mL of phosphate-buffered saline solution into 5 mL polypropylene tubes. Mucosa samples were homogenized using a tissue homogenizer (Tissuemiser, Thermo Fisher Scientific Inc., Rockford, IL, USA) for 30 s on ice and transferred to a new 2 mL microcentrifuge tube for centrifugation for 15 min at 14,000×*g* (MiniSpin plus; Eppendorf AG, Hamburg, Germany) as described by Holanda et al [22]. The supernatant was divided into 6 sets of 0.15 mL into polypropylene tubes and stored at −80°C for further analysis.

### Intestinal health makers

The concentrations of total protein, tumor necrosis factor-alpha (TNF-α), interleukin-8 (IL-8), immunoglobulin A (IgA), and immunoglobulin G (IgG) in proximal and distal jejunal mucosa were analyzed by the colorimetric method and the absorbance was measured on a plate reader (Synergy HT; BioTek Instruments, Winooski, VT, USA).

The concentration of total protein was analyzed using Pierce BCA Protein Assay Kit (#23225; Thermo Fisher Scientific, USA) as described by Holanda et al [22]. Mucosa samples were diluted (1:50) to reach the working range of 20 to 2,000 μg/mL. The absorbance was measured at 562 nm. The concentration of total protein was calculated by the standard curve and used to normalize the concentrations of TNFα, IL-8, IgA, and IgG.

The concentration of TNF-α was analyzed using the Porcine TNF-α Immunoassay Kit (#PTA00; R&D Systems, Minneapolis, MN, USA) as described by Duarte et al [26]. The working range of standards was 0 to 1,500 pg/mL. The absorbance was measured at 450 nm and corrected with 570 nm. The concentration of TNF-α was calculated by the standard curve and described as pg/mg of protein.

The concentration of IL-8 was analyzed using the Porcine IL-8/CXCL8 Immunoassay Kit (#P8000; R&D Systems, USA) following Moita et al [27]. Mucosa samples were diluted (1:6) to reach the working range of standards from 0 to 4,000 pg/mL. The absorbance was measured at 450 nm and corrected with 570 nm. The concentration of IL-8 was calculated by the standard curve and described as ng/mg of protein.

The concentration of IgA was analyzed using the pig ELISA kit (#E101–102; Bethyl Laboratories, Montgomery, TX, USA) following Holanda et al [22]. Mucosa samples were diluted (1:1,500) to reach the working range of standards from 15.6 to 1,000 ng/mL. The absorbance was measured at 450 nm and corrected with 540 nm. The concentration of IgA was calculated by the standard curve and described as μg/mg of protein.

The concentration of IgG was analyzed using the pig ELISA kit (#E101–104; Bethyl Laboratories, USA) following Duarte et al [26]. Mucosa samples were diluted (1:3,600) to reach the working range of standards from 7.8 to 500 ng/mL. The absorbance was measured at 450 nm and corrected with 570 nm. The concentration of IgG was calculated by the standard curve and described as μg/mg of protein.

### Apparent ileal digestibility

Frozen ileal digesta samples were freeze dried (24D×48, Virtis, Gardiner, NY, USA). Then, phase 2 diets and dried ileal digesta were ground to fine powder form (Method 934.01, AOAC, 2006). The concentration of titanium dioxide in diets and digesta was measured following Myers et al [28]. The dry matter (DM) was measured by following Passos et al [28]. Gross energy (GE) was measured using bomb calorimeter (Parr 6200; Parr instrument company, Moline, IL, USA). The crude protein (CP) was measured in the diets and digesta by using LECO CN-2000 Nitrogen Analyzer (LECO Corporation, St. Joseph, MI, USA) following (AOAC method 990.03) [30]. Ether extract (EE) was measured by using a Soxhlet extraction method (AOAC method 920.39) [30].

Apparent ileal digestibilities of DM, GE, CP, and EE were calculated using the titanium dioxide concentration in diets and digesta following Chen et al [31]. The AID was calculated with the following equation:


$$\text{AID}\%=[1-({\text{TiO}}_{2}\;\text{in diet}/{\text{TiO}}_{2}\;\text{in digesta})\times (\text{nutrient in digesta}/\text{nutrient in diet})]\times 100$$

### Statistical analysis

Data were analyzed using the MIXED procedure in SAS 9.3 (SAS Inc, Cary, NC, USA). Dietary treatments were defined as fixed effects. Initial BW and sex were considered as blocks and defined as random effects. Outliers were defined as data greater than the 75th percentile+1.5×the interquartile range and smaller than the 25th percentile–1.5×the interquartile range and removed from analysis. One pig from treatment YL1.5 was removed for analysis of AID of CP and EE due to insufficient digesta. The least squares mean comparisons with the general linear model procedure was used to calculate mean values for all treatments. The least significant difference test (PDIFF) was used for a pairwise comparison among dietary treatments. The analysis of fecal score data was performed by using Kruskal-Wallis Test with Dwass, Steel, Critchlow-Fligner method option for pairwise two-sided multiple comparisons following Guo et al [25]. Statistical significance was p<0.05 and 0.05≤p<0.10 was considered as a tendency.

## RESULTS

**Figure 1:**
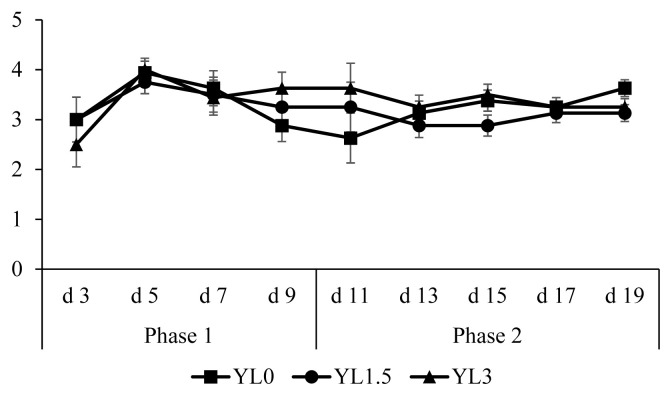
Fecal score of pigs fed diet with *Yarrowia lipolytica*. Fecal score: (1) very firm stool, (2) normal firm stool, (3) moderately loose stool, (4) loose, watery stool and (5) very watery stool with no shape. The dietary treatments consisted of a basal diet supplemented with *Yarrowia lipolytica* at 0.0%, 1.5%, and 3.0% as YL0, YL1.5 and YL3, respectively.

Initial BW was 7.2±0.6 kg and did not differ among treatments. Overall ADG, ADFI, and G:F were 252±37 g, 421±52 g, and 0.60±0.04, respectively and these were not affected by the supplementation of *Yarrowia lipolytica* at 1.5% and 3.0% in diets (Table 3). There were no difference in fecal scores among the treatments (Figure 1). Supplemental *Yarrowia lipolytica* at 1.5% and 3.0% in diets did not affect the concentrations of intestinal immune markers, including TNF-α (0.67±0.12 and 2.15±0.40 pg/mg), IL-8 (0.66±0.07 and 1.02±0.22 ng/mg), IgA (1.58±0.17 and 1.51±0.21 μg/mg) and IgG (4.39±0.33 and 4.95±0.67 μg/mg) in proximal and distal jejunal mucosa (Table 4). Pigs fed diets with 3.0% *Yarrowia lipolytica* had lower (p<0.05) AID of DM (40.2% to 55.0%), GE (44.0% to 57.5%), and EE (50.8% to 66.9%) than pigs fed diets with 0.0% *Yarrowia lipolytica* (Table 5). Pigs fed diets with 3.0% *Yarrowia lipolytica* tended to have lower (p = 0.074) AID of CP (52.1% to 66.1%) than pigs fed 0.0% *Yarrowia lipolytica*. Pigs fed diets with 1.5% *Yarrowia lipolytica* had higher (p<0.05) AID of GE (56.5% to 44.0%) and EE (62.3% to 50.8%) than pigs fed diets with 3.0% *Yarrowia lipolytica*. However, pigs fed diets with 1.5% *Yarrowia lipolytica* did not affect AID of DM (52.5% to 55.0%), GE (56.5% to 57.5%), CP (61.8% to 66.1%), and EE (62.3% to 66.9%) in diets compared to pigs fed diets with 0.0% *Yarrowia lipolytica*.

## DISCUSSION

This study showed that supplementation of Y*arrowia lipolytica* at a range of 1.5% and 3.0% is an effective fat supplement in nursery diets. Supplementing nursery diets with *Yarrowia lipolytica* at 1.5%, replacing 0.8% poultry fat and 0.45% blood plasma, did not affect the growth performance, intestinal health and nutrient digestibility. Supplementing *Yarrowia lipolytica* at 3.0% replacing 1.6% poultry fat and 0.9% blood plasma did not adversely affect the growth performance of pigs, whereas it reduced the AID of DM, GE, CP, and EE. The current results are in agreement with previous studies that reported the supplementation of yeasts at 30% in fish diets [13] and at 1.6×107 colony-forming unit/g of nursery diets [32] reduced the apparent digestibility of nutrients but did not affect the overall growth performance. Conversely, Czech et al [33] reported that supplementation of 3.0% of *Yarrowia lipolytica* replacing soybean meal enhanced the growth performance of nursery pigs from 10 to 32 kg. A reduction of nutrient digestibility with 3.0% of *Yarrowia lipolytica* without affecting the growth performance observed in this study is probably due to the bioactive compounds from yeasts including oleic acid, β-glucans and mannans that play important roles on microbiota modulation and immune system [34,35].

The decreased nutrient digestibility observed in this study may be due to the complex structures from yeast cell walls that have negative effects on nutrient digestibility [22]. In this study, *Yarrowia lipolytica* was added to diets by replacing up to 1.6% poultry fat and 0.9% blood plasma. Considering that the *Yarrowia lipolytica* was not processed to lyse the cell wall prior the study, the cell wall may have partially protected the fat from digestion which may have contribute to reduce the EE digestibility [36]. Additionally, the protein in the *Yarrowia lipolytica* used in this study may have lower digestibility compared to blood plasma and consequently reduced the CP digestibility in feed with 3.0% *Yarrowia lipolytica*. The yeast cell wall are fermented by the intestinal microbiota in the large intestine to produce short-chain fatty acids (SCFA) and, consequently, release cell components in the large intestine [37]. In addition to the cell wall compounds, the undigested nutrients are fermented by the intestinal microbiota along the intestine affecting the production SCFA and consequently affecting the energy utilization [38]. The majority microbial fermentation occurs in the cecum-colon portion [39], therefore, the SCFA produced during fermentation in the large intestine was not counted in the analysis of AID, although they could affect the growth performance even with reduced AID of nutrients [40]. Yeast cell wall is known for their effects on microbiota modulation of intestinal microbiota [37]. Kiros et al [41] showed that the increased relative abundance of *Prevotella*, an effective producer of SCFAs, in pigs fed diets with yeast had positive correlations with growth performance by increasing energy utilization. In addition, dietary mannans and β-glucans have been related to increased relative abundance of butyrate-producing bacteria including *Clostridium* [35] and *Faecalibacterium prausnitzii* [42]. Besides the energy metabolism, butyric acid is known as an important compound for the intestinal health status [43].

It is well known that supplementing blood plasma in nursery diets improves the growth performance of pigs [20]. Besides the highly digestible protein, 20% of protein in blood plasma is IgG that inhibit the adherence of antigens in the intestinal mucosa, enhancing the immune response and, consequently improving the growth performance [44]. *Yarrowia lipolytica* has a high concentration of oleic acid, which possesses anti-inflammatory and anti-oxidative properties [15] and, consequently, would enhance the health and growth performance of pigs. Similar to the immune modulating function of dietary blood plasma, β-glucans and mannans present in yeast cell wall are beneficial to animal growth and health [19]. The first mechanism of β-glucan and mannans is that both can bind to the carbohydrate receptors on pathogenic bacteria preventing infection by inhibiting the adherence of pathogens to the intestinal mucosa [32]. Secondly, they can be bind by receptors on intestinal epithelial cells, including M cells, macrophages and dendritic cells and, consequently activate the innate response to release cytokines and adoptive response [45]. Following the innate response, activated intestinal epithelial cells stimulate Th-1 cells to reduce the production of pro-inflammatory cytokines, such as TNF-α, IL-6, and IL-8 [34]. Li et al [18] described that the uptake of β-glucans from *Saccharomyces cerevisiae* at 50 mg/kg in nursery increased the release of the anti-inflammatory cytokine, IL-10, to inhibit the overproduction of proinflammatory cytokines, TNF-α and IL-6 in plasma under *Escherichia coli* challenge. In addition, supplementing *Saccharomyces cerevisiae* in pigs [46] enhanced immune system by promoting the concentrations of IgA and IgG in blood and resulted in improved growth performance. However, the immune markers measured in this study were not adversely affected by supplementing *Yarrowia lipolytica* up to 3.0% indicating that the bioactive components from *Yarrowia lipolytica* may have similar effects as those expected from blood plasma [20] for maintaining animal growth and health.

In conclusion, supplementing *Yarrowia lipolytica* at 1.5% in diets successfully reduced 0.8% of poultry fat and 0.45% blood plasma, without affecting growth performance, intestinal health and nutrient digestibility in diets fed to nursery pigs. Moreover, supplementing *Yarrowia lipolytica* at 3.0% replacing 1.6% poultry fat and 0.9% blood plasma in diets did not cause an adverse effect on growth performance and intestinal health possibly due to the benefits from oleic acid, β-glucans and mannans in *Yarrowia lipolytica*, whereas it reduced digestibility of nutrients in diets fed to nursery pigs.

## Figures and Tables

**Table 1:** The nutrient composition of *Yarrowia lipolytica* and common fat supplements [23]

Items	*Yarrowia lipolytica* ^1)^
Gross energy (kcal/kg)	7,996
FA^2)^ in EE (%)	
Myristic acid, C14:0	0.1
Palmitic acid, C16:0	12.5
Palmitoleic acid, C16:1	4.0
Stearic acid, C18:0	16.1
Oleic acid, C18:1	52.3
Linoleic acid, C18:2n6	9.6
DHA, C22:6n3	2.7
Unsaturated FA in total fat	68.8
Crude protein (%)	9.7
Amino acids (%)^3)^	
Histidine	0.25
Isoleucine	0.40
Leucine	0.71
Lysine	0.62
Methionine	0.14
Phenylalanine	0.39
Threonine	0.42
Tryptophan	0.15
Valine	0.47

FA, fatty acids; EE, ether extract; DHA, docosahexaenoic acid.

1) *Yarrowia lipolytica* contain 9.7% CP and 72.7% fat in the total yeast.

2) Based on analyzed composition using gas chromatography/mass spectrometry (GC/MS) method.

3) Amino acid profile is based on Michalik et al [24].

**Table 2:** Feed formulation and nutrient composition of diets with *Yarrowia lipolytica*

Ingredient	Phase 1^1)^			Phase 2^1)^		
	
	YL0	YL1.5	YL3	YL0	YL1.5	YL3
Feedstuff (%)						
Yellow dent corn, ground	42.8	42.5	42.2	49.7	49.3	49.0
Whey permeate	24.0	24.0	24.0	15.0	15.0	15.0
Soybean meal, 48% CP	20.0	20.0	20.0	23.0	23.0	23.0
Poultry meal	3.00	3.00	3.00	3.00	3.00	3.00
Fish meal	2.00	2.00	2.00	0.00	0.00	0.00
* * *Yarrowia lipolytica*	0.00	1.50	3.00	0.00	1.50	3.00
Poultry fat	2.15	1.36	0.57	1.98	1.19	0.40
Blood plasma	2.90	2.45	2.00	4.10	3.65	3.20
L-lysine HCl	0.72	0.75	0.77	0.43	0.46	0.48
DL-methionine	0.31	0.33	0.33	0.18	0.20	0.20
L-threonine	0.27	0.28	0.29	0.12	0.13	0.14
L-tryptophan	0.05	0.06	0.06	0.00	0.01	0.01
Salt	0.25	0.25	0.25	0.25	0.25	0.25
Dicalcium phosphate	0.65	0.65	0.65	0.85	0.85	0.85
Limestone	0.70	0.70	0.70	0.85	0.85	0.85
Vitamin premix^2)^	0.03	0.03	0.03	0.03	0.03	0.03
Mineral premix^3)^	0.15	0.15	0.15	0.15	0.15	0.15
Titanium dioxide	0.00	0.00	0.00	0.40	0.40	0.40
Calculated composition						
DM (%)	90.6	90.7	90.8	90.2	90.3	90.3
ME (kcal/kg)	3,401	3,401	3,401	3,401	3,401	3,401
CP (%)	20.3	20.0	19.6	21.1	20.9	20.5
SID lysine (%)	1.50	1.50	1.50	1.35	1.35	1.35
SID methionine+cysteine (%)	0.83	0.84	0.83	0.74	0.76	0.74
SID tryptophan (%)	0.25	0.26	0.25	0.23	0.24	0.23
SID threonine (%)	0.88	0.88	0.88	0.79	0.79	0.79
Ca (%)	0.85	0.85	0.85	0.80	0.81	0.80
STTD P (%)	0.46	0.46	0.45	0.42	0.42	0.41
Analyzed composition (%)						
DM	90.4	90.3	90.4	90.4	89.7	90.2
CP	22.36	21.87	22.34	22.69	23.35	23.00
EE	4.68	4.54	4.49	4.90	4.47	4.48

CP, crude protein; DM, dry matter; ME, metabolizable energy; SID, standardized ileal digestible; STTD, standardized total tract digestible; EE, ether extract.

1) The dietary treatments consisted of a basal diet supplemented with *Yarrowia lipolytica* at 0.0%, 1.5%, and 3.0% as YL0, YL1.5 and YL3, respectively.

2) The vitamin premix provided the following per kilogram of complete diet: 6,613.8 IU of vitamin A as vitamin A acetate, 992.0 IU of vitamin D_3_, 19.8 IU of vitamin E, 2.64 mg of vitamin K as menadione sodium bisulfate, 0.03 mg of vitamin B_12_, 4.63 mg of riboflavin, 18.52 mg of D-pantothenic acid as calcium pantothenate, 26.45 mg of niacin and 0.07 mg of biotin.

3) The trace mineral premix provides the following per kilogram of complete diet: 33.0 mg Mn as manganous oxide, 109.5 mg of Fe as ferrous sulfate, 109.5 mg of Zn as zinc sulfate, 16.5 mg of Cu as copper sulfate, 0.3 mg of I as ethylenediamine dihydroiodide and 0.3 mg of Se as sodium selenite.

**Table 3:** Growth performance of pigs fed diet with *Yarrowia lipolytica*

Item	Treatment^1)^			SEM	p-value

	YL0	YL1.5	YL3		
BW (kg)					
Initial	7.1	7.2	7.2	0.6	0.979
d 10	7.9	8.3	8.3	1.0	0.584
d 21	12.1	12.9	12.4	1.4	0.563
ADG (g)					
d 0 to 10	73	112	113	41	0.472
d 10 to 21	387	419	370	53	0.636
Overall	237	273	248	37	0.542
ADFI (g)					
d 0 to 10	164	186	208	34	0.343
d 10 to 21	691	616	625	71	0.244
Overall	433	411	419	52	0.741
G:F					
d 0 to 10	0.45	0.64	0.64	0.08	0.175
d 10 to 21	0.56	0.68	0.63	0.04	0.104
Overall	0.55	0.66	0.59	0.04	0.165

SEM, standard error of mean; BW, body weight; ADG, average daily gain; ADFI, average daily feed intake; G:F, gain to feed ratio.

1) The dietary treatments consisted of a basal diet supplemented with *Yarrowia lipolytica* at 0.0%, 1.5%, and 3.0% as YL0, YL1.5, and YL3, respectively.

**Table 4:** Intestinal health markers in the jejunal mucosa of pigs fed diets with *Yarrowia lipolytica*

Item (%)	Treatment^1)^			SEM	p-value

	YL0	YL1.5	YL3		
Proximal^2)^ jejunal mucosa, concentration/mg of protein					
TNF-α (pg)	0.64	0.77	0.61	0.12	0.570
IL-8 (ng)	0.62	0.75	0.62	0.07	0.300
IgA (μg)	1.45	1.52	1.55	0.17	0.918
IgG (μg)	4.45	4.04	4.79	0.33	0.329
Distal jejunal mucosa, concentration/mg of protein					
TNF-α (pg)	2.59	1.75	2.13	0.40	0.349
IL-8 (ng)	1.12	0.79	1.16	0.22	0.370
IgA (μg)	1.08	1.07	1.47	0.21	0.314
IgG (μg)	4.64	4.21	5.99	0.67	0.174

SEM, standard error of mean; TNF-α, tumor necrosis factor alpha; IL-8, interleukin-8; IgA, immunoglobulin A; IgG, immunoglobulin G.

1) The dietary treatments consisted of a basal diet supplemented with *Yarrowia lipolytica* at 0.0%, 1.5%, and 3.0% as YL0, YL1.5, and YL3, respectively.

2) Proximal: 1.5 m after the pyloric duodenal junction, distal: 1.5 m before the ileocecal junction.

**Table 5:** Apparent ileal digestibility of nutrients in diets with fed to pigs *Yarrowia lipolytica*

Item (%)	Treatment^1)^			SEM	p-value

	YL0	YL1.5	YL3		
DM	55.0^a^	52.5^ab^	40.2^b^	4.1	0.045
GE	57.5^a^	56.5^a^	44.0^b^	3.9	0.047
CP	66.1^A^	61.8^AB^	52.1^B^	4.2	0.084
EE	66.9^a^	62.3^a^	50.8^b^	3.4	0.011

SEM, standard error of mean; DM, dry matter; GE, gross energy; CP, crude protein; EE, ether extract.

1) The dietary treatments consisted of a basal diet supplemented with *Yarrowia lipolytica* at 0.0%, 1.5%, and 3.0% as YL0, YL1.5, and YL3, respectively.

a,b Within a row, means without a common superscripts letter are significant different (p<0.05).

A,B Within a row, means without a common superscripts letter are tended to be different (p<0.10).
